# Editorial: 15 years of frontiers in psychiatry – wellbeing and society

**DOI:** 10.3389/fpsyt.2026.1993698

**Published:** 2026-10-01

**Authors:** Wulf Rössler, Marco Grados

**Affiliations:** 1 Department of Psychiatry, Psychotherapy and Psychosomatics, University of Zurich, Zurich, Switzerland; 2 School of Medicine, Johns Hopkins University, Baltimore, MD, United States

**Keywords:** coercion, contextual diagnosis, gender-sensitive, prevention, psychosocial

As Frontiers in Psychiatry celebrates its 15th anniversary, this Research Topic critically reflects on some pivotal milestones, paradigm shifts, and debates that have defined psychiatric research since then.

From a total of 18 submissions, nine articles were selected for publication. While this collection naturally cannot claim to be representative of the entire breadth of psychiatric research, these contributions strategically highlight key directions that have gained significant influence. Notably, the selected articles focus predominantly on psychosocial, systemic, and ethical dimensions, rather than biological or neuroscientific mechanisms. Together, they illustrate the wide spectrum of contemporary psychosocial themes in psychiatry, ranging from complex systemic interactions to rights-based frameworks, charting a course for future breakthroughs aimed at advancing mental wellbeing at both societal and individual levels.

A dominant theme across this collection is the urgent need to expand psychiatry’s lens beyond the individual. (Ajdacic-Gross et al.) argue, that while complexity is increasingly recognized in mental disorder research, current approaches risk generating findings without transformative breakthroughs. They propose a multisystem perspective that embraces the heterogeneity of systems, processes, and interactions inherent in mental health, drawing on examples from suicide and neurodevelopmental disorder research.

Similarly, (Batstra and Frances) contend that the dramatic rise in psychiatric diagnoses since the DSM-III has diverted attention from the social contexts in which mental suffering occurs. They advocate for supplementing individual diagnosis with “context diagnosis” to address the root causes of distress.

This contextual shift is further explored through geopolitical and economic determinants. (Bhugra et al.) introduce geo-psychiatry as a framework for understanding how “poly-crisis - the convergence of armed conflict, climate change, and forced migration - creates psychiatric vulnerabilities that transcend borders. They argue that patient-centered interventions alone are insufficient without addressing these structural inequalities.

In a complementary analysis (Kalinowski & Rössler) demonstrate that women face a “double risk” in contexts of economic inequality: higher exposure to disadvantage and greater vulnerability to its mental health effects, particularly regarding depression. Their multivariate meta-analysis underscores the necessity of gender-sensitive approaches in policy and research.

The tension between established practices and emerging ethical paradigms is sharply evident in discussions of coercion and legislation. (Jaeger and Theodoridou) analyze the Swiss professional discourse on coercive measures, revealing a broad consensus on the ethical problems of coercion but significant divergence on normative goals - ranging from reduction to abolition.

This debate mirrors the global paradigm shift documented by (Xue and Chen), who compare WHO guidelines from 2006 and 2023. They highlight a decisive transition from biomedical approaches accepting involuntary treatment toward a human rights-based framework that rejects coercion and promotes supported decision-making, albeit with significant implementation challenges.

Finally, the collection addresses the critical need for prevention and the long-term impacts of systemic failures. (El Alaoui) proposes a shift from “run-to-failure” models to condition-based care, presenting a multi-domain framework for preventive psychiatry that leverages objective monitoring of sleep, nutrition, and inflammatory markers.

The consequences of reactive models are illustrated by (Chen et al.), whose longitudinal data show increasing psychological distress among healthcare workers exposed to biological disasters over two decades, emphasizing the need for sustained, system-level support. Complementing this, (Koh and Lee) quantify the profound impact of mental disorders on health-related quality of life in Korea, showing a 71% reduction in EQ-5D scores, while highlighting self-rated health as a modifiable protective factor.

Together, these articles underscore that the next decade of psychiatric research must integrate multisystem complexity, social context, human rights, and preventive care. By deliberately prioritizing psychosocial and structural determinants over purely biological inquiries, this collection demonstrates the rich variety of approaches available to address the root causes of mental distress. Moving beyond reactive, individual-focused interventions allows psychiatry to engage more deeply with the geopolitical and societal forces shaping mental health, ultimately fostering resilience and wellbeing on a global scale.

